# Navigation is associated with lower risk of neurological injury and transfusion in pediatric idiopathic scoliosis surgery

**DOI:** 10.1007/s43390-025-01140-w

**Published:** 2025-06-29

**Authors:** Vivien Chan, Suhas Etigunta, Adeesya Gausper, Andy M. Liu, Kenneth D. Illingworth, Grant D. Hogue, Daniel J. Hedequist, David L. Skaggs

**Affiliations:** 1 Cedars Sinai Spine, Cedars Sinai Medical Center, 444 S San Vicente Blvd, Ste 900, Los Angeles, CA 90048 USA; 2 Boston Children’s Hospital, Harvard University, Boston, MA USA

**Keywords:** Spinal navigation, Neurological injury, Transfusion, Reoperation, Operative time, Idiopathic scoliosis, Pediatric

## Abstract

**Background:**

Spinal navigation has been shown to improve accuracy with pedicle screw placement and reduce complications in adult spine patients. It remains understudied in the pediatric spine population.

**Purpose:**

The purpose of this study was to assess the impact of spinal navigation on rates of neurological injury, allogeneic transfusion, and reoperation in pediatric idiopathic scoliosis.

**Methods:**

This was a retrospective cohort study using the NSQIP pediatric database, years 2016–2022. Patients were included in this study if they were under 18 years of age and received posterior spinal fusion with seven or more surgical levels for idiopathic scoliosis. Anterior approaches were excluded from the study. The study cohort was divided into (1) no navigation cohort and (2) navigation cohort. The primary outcome was rate of postoperative neurological injury. Secondary outcomes were 30 day reoperation, allogeneic blood transfusion, and operative time. Rates of neurological injury, reoperation, and allogeneic transfusion were compared using Chi-square test. Operative time was compared using Student’s T test. Logistic regression analyses were performed to determine the association between use of spinal navigation and the outcomes of interest.

**Results:**

There were 22,384 patients included in this study. Mean age was 14.4 years. Spinal navigation was used in 1879 (8.4%). Spinal navigation was associated with a reduced rate of postoperative neurological injury (no navigation: 1.2% vs. navigation: 0.6%, *p* = 0.02). The navigation cohort had a lower rate of allogeneic transfusion (no navigation: 12.2% vs. navigation: 8.4%, *p* < 0.001). There was no difference in 30 day reoperation rate (no navigation: 1.4% vs. navigation: 1.5%, *p* = 0.56). The navigation cohort had longer operative time (no navigation: 4.6 h vs. navigation: 5.0 h, *p* < 0.001). In the multivariable regression analysis, use of spinal navigation was associated with reduced odds of postoperative neurological injury (OR = 0.51, *p* = 0.03) and allogeneic transfusion (OR = 0.68, *p* < 0.001).

**Conclusion:**

Spinal navigation was associated with significantly decreased rates of postoperative neurological injury and allogeneic transfusion in pediatric idiopathic scoliosis surgery, with an average of 0.4 h longer operative time.

## Introduction

Spinal navigation utilizes computed tomography (CT) or three-dimensional fluoroscopy to create a three-dimensional map to help guide surgeons intraoperatively [1]. Three-dimensional visualization of the anatomy can be especially useful in scoliosis where the anatomy can deviate significantly from normal anatomy or in cases where key anatomy for screw placement is difficult to visualize [2, 3]. Malpositioned screws can result in damage to nerve roots, the spinal cord, major vessels, and organs, resulting in serious complications [4, 5]. Navigation was developed to improve pedicle screw placement accuracy and minimize iatrogenic morbidity [1, 6].

Use of spinal navigation systems in adult spine surgery started in the 1990s and has become a mainstay in adult spine surgery [4]. Previous studies have demonstrated positive outcomes related to the use of navigation in adult patients treated for degenerative spine disease [6]. However, literature on the use of navigation in pediatric scoliosis remains limited [6]. The purpose of this study was to assess the impact of navigation on rates of neurological injury, allogeneic transfusion, and reoperation in pediatric idiopathic scoliosis.

## Methods

### Participants and study design

This was a retrospective cohort study using the American College of Surgeons National Surgical Quality Improvement Program (NSQIP) Pediatric database, years 2016–2022. NSQIP is a nationally validated, risk-adjusted, outcomes-based program that measures the quality of surgical care. In each participating hospital, a trained surgical clinical reviewer collects preoperative through 30 day postoperative data on randomly selected patients. Data are anonymized and entered online in a secure, web-based platform available for open access by request from a participating center.

Patients were included in this study if they were (1) under 18 years of age, (2) underwent posterior arthrodesis with seven or more surgical levels (CPT 22802, 22,804), and (3) had a diagnosis of idiopathic scoliosis. Anterior arthrodesis and anterior osteotomies were excluded in this study. Congenital, syndromic, and neuromuscular scoliosis were excluded from this study. The study cohort was divided into (1) no navigation cohort and (2) navigation cohort. Use of navigation was defined using CPT 61783.The NSQIP Pediatric database excludes: (1) patients 18 years of age or older, (2) cases involving hyperthermic intraperitoneal chemotherapy, (3) ASA score of 6, (4) concurrent case by different surgical team under the same anesthetic, (5) multiple cases within 30 days, (6) transplant cases, and (7) trauma and abuse cases. The primary outcome of this study was the rate of postoperative neurological injury. Secondary outcomes included rate of 30 day reoperation, rate of perioperative allogeneic transfusion, and operative time (h). The NSQIP pediatric database collects data on perioperative autogenic transfusion through cell salvage and allogeneic transfusion. In this study, perioperative allogeneic transfusion was defined as receiving intraoperative or postoperative allogeneic transfusion.

### Statistical analysis

Descriptive statistics were used to characterize patient and surgical characteristics. Continuous variables were reported as means with standard deviation and categorical variables were reported as counts and proportions. The rates of postoperative neurological injury, perioperative allogeneic transfusion, and 30 day reoperation and mean operative time for each surgical year were determined. The rates of postoperative neurological injury, perioperative allogeneic transfusion, and 30 day reoperation were determined as proportions for each cohort and compared using Chi-square test. The number needed to treat (NNT) was determined for significant outcomes. Operative time (h) was determined as means with standard deviation for each cohort and compared using Student’s t test. Neurological injury was categorized as (1) spinal cord injury and (2) nerve root/plexus/cauda equina injury and rates of these injuries were reported for each cohort. The proportion of navigation cases for each year from 2016 to 2022 were determined.

Binary multivariable logistic regression analyses were used to determine the association between the use of navigation and odds of postoperative neurological injury, perioperative allogeneic transfusion, and 30 day reoperation. All regression models were adjusted for age in years, sex, ASA score, surgical year, and number of surgical levels (7–12 levels vs. > 12 levels). These variables were chosen a priori based on a literature search and clinical significance. The alpha value for significance was 0.05. Listwise deletion was used for missing data for complete case analysis. Statistical analysis was performed using IBM SPSS Statistics version 29.0.2.0.

## Results

### Patient and surgical characteristics

**Fig. 1:**
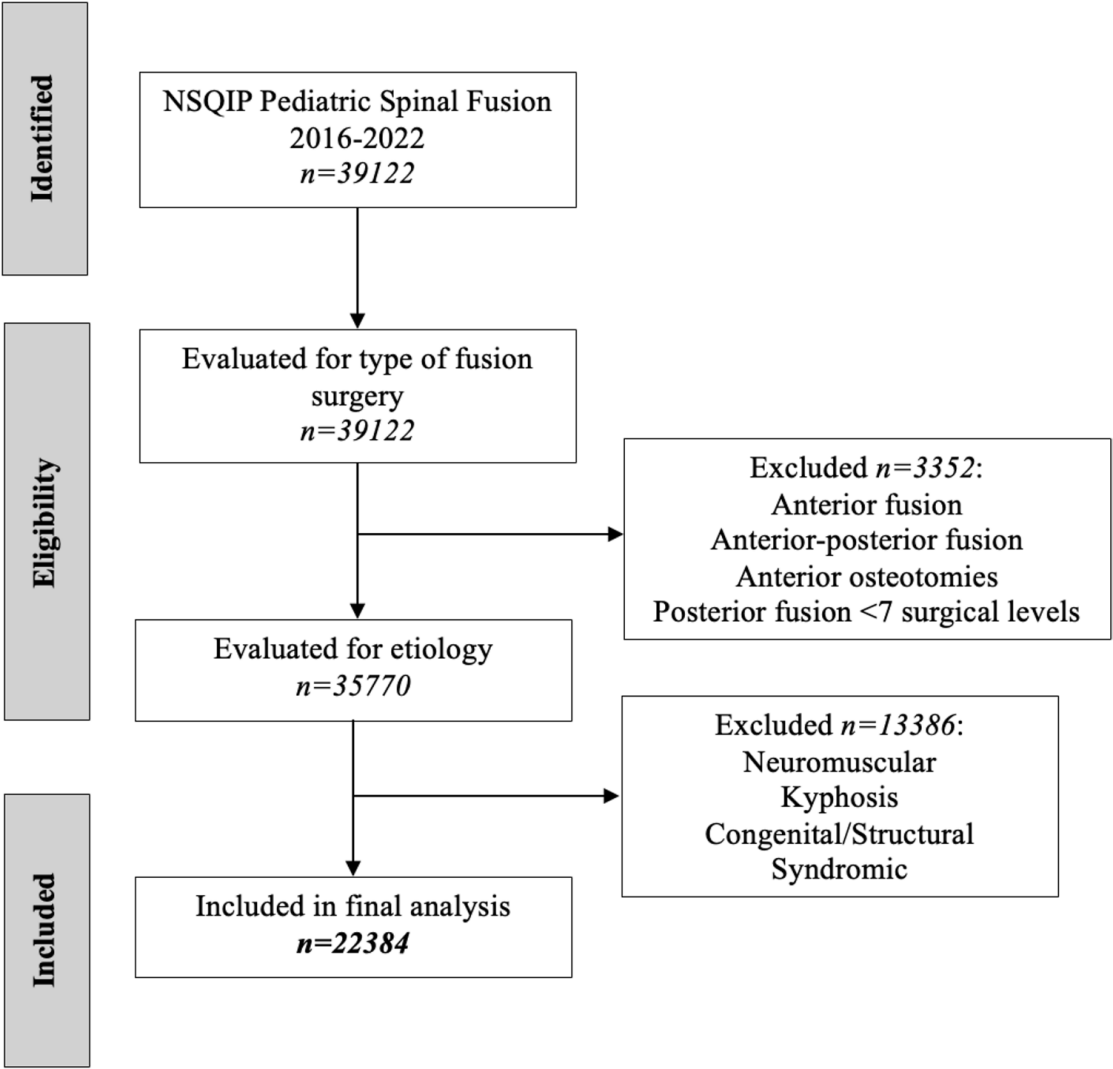
CONSORT flowchart diagram

A total of 22,384 patients were included in this study, with 20,505 in the no navigation cohort and 1879 in the navigation cohort (Fig. 1). The mean age of the entire cohort was 14.4 ± 1.9 years, and 76.7% (*n* = 17,173) were female (Table 1). Of the 22,384 patients, 23.4% (*n* = 5284) had an ASA score of 1, 64.6% (14,452) an ASA score of 2, 11.7% (*n* = 2622) an ASA score of 3, and 0.3% (*n* = 62) an ASA score of 4. 71.9% (*n* = 16,105) of the patients had 7–12 surgical levels and 28.1% (*n* = 6279) had > 12 surgical levels. There was a significant difference in the proportion of female patients (*p* = 0.006) and ASA score (*p* < 0.001) between the two cohorts (Table 1). The proportion of cases performed with navigation increased from 2016 to 2022, with 2.1% (*n* = 44) performed in 2016 and 15.6% (*n* = 610) in 2022 (Table 2). In a comparison of the baseline characteristics, the no navigation cohort had more female patients and more patients who had an ASA score of 1. The navigation cohort had more patients who had an ASA score of 2 and 3. There was no significant difference between the two cohorts in age or number of surgical levels.

**Table 1:** Patient and surgical characteristics in the “no navigation” and “navigation” cohorts and a comparison of baseline differences between the two cohorts

	No navigation (*n* = 20,505)	Navigation (*n* = 1879)	*p* value
Age (years)	14.4 ± 1.9	14.4 ± 2.0	0.926
Female	15,780 (77.0%)	1393 (74.1%)	0.006*
ASA score			
1	4,957 (24.2%)	291 (15.5%)	< 0.001*
2	13,127 (64.0%)	1325 (70.5%)	
3	2366 (11.5%)	256 (13.6%)	
4	55 (0.3%)	7 (0.4%)	
Number of surgical levels			
7–12 levels	14,771 (72.0%)	1334 (71.0%)	0.336
> 12 levels	5734 (28.0%)	545 (29.0%)

**Table 2:** Proportion of navigation cases by surgical year

Year	Navigation cases % (*n*)
2016	2.1% (44/2138)
2017	3.9% (96/2443)
2018	5.2% (159/3029)
2019	8.3% (293/3511)
2020	7.8% (261/3360)
2021	10.4% (416/3983)
2022	15.6% (610/3920)

### Postoperative neurological injury

The rate of postoperative neurological injury for the entire study cohort was 1.1% (*n* = 248). The rates of postoperative neurological injury for each surgical year from 2016 to 2022 are reported in Table 3. The rates ranged from 0.9% to 1.6%. The navigation cohort had a significantly lower rate of postoperative neurological injury (no navigation: 1.2% vs. navigation: 0.6%, *p* = 0.02) (Table 4). The NNT to prevent one neurological injury was 167. The rates of spinal cord injury for the no navigation cohort and navigation cohort were 0.30% (*n* = 60) and 0.05% (*n* = 1), respectively (Table 5). The rates of nerve root/plexus/cauda equina injury for the no navigation cohort and navigation cohort were 0.75% (*n* = 153) and 0.43% (*n* = 8), respectively. In the multivariable logistic regression analysis, the use of navigation was associated with reduced odds of postoperative neurological injury (OR 0.51 [95% CI 0.28–0.94], *p* = 0.03) (Table 6). An ASA score of 4 was associated with higher odds of postoperative neurological injury (OR 6.43 [95% CI 2.24–18.45], *p* < 0.001). Age in years, female sex, surgical year, and number of surgical levels were not associated with postoperative neurological injury.

**Table 3:** Rates of allogeneic transfusion, neurological injury, and 30 day reoperation and mean operative time by surgical year

	2016	2017	2018	2019	2020	2021	2022
Postoperative Neurological injury	1.6% (*n* = 35)	0.9% (*n* = 23)	0.9% (*n* = 28)	1.0% (*n* = 35)	1.4% (*n* = 47)	1.0% (*n* = 41)	1.0% (*n* = 39)
Allogeneic transfusion	16.0% (*n* = 342)	13.9% (*n* = 340)	11.2% (*n* = 338)	12.5% (*n* = 440)	10.0% (*n* = 336)	11.7% (*n* = 467)	10.3% (*n* = 405)
30 day reoperation	1.4% (*n* = 31)	1.2% (*n* = 30)	1.6% (*n* = 47)	1.2% (*n* = 43)	1.2% (*n* = 39)	1.7% (*n* = 66)	1.4% (*n* = 56)
Operative time (h)	4.51 ± 1.59	4.47 ± 1.59	4.50 ± 1.54	4.56 ± 1.46	4.52 ± 1.44	4.70 ± 1.49	4.78 ± 1.52

**Table 4:** Comparison of the rates of postoperative neurological injury, allogeneic transfusion, and 30 day reoperation. Comparison of operative time and allogeneic transfusion volume

	No navigation	Navigation	*X*^2^	*p* value
Postoperative neurological injury	1.2% (*n* = 237)	0.6% (*n* = 11)	5.11	0.02*
Allogeneic transfusion	12.2% (*n* = 2510)	8.4% (*n* = 158)	24.08	< 0.001*
30 day reoperation	1.4% (*n* = 283)	1.5% (*n* = 29)	0.33	0.56

	No navigation	Navigation	t	*p* value
Operative time (h)	4.56 ± 1.51	4.97 ± 1.55	-11.16	< 0.001*

**Table 5:** Characterization of postoperative neurological injury in the no navigation cohort and navigation cohort

	No navigation	Navigation
Spinal cord injury	0.30% (*n* = 60)	0.05% (*n* = 1)
Nerve root/plexus/ cauda equina injury	0.75% (*n* = 153)	0.43% (*n* = 8)

**Table 6:** Multivariable regression analysis on spinal navigation and postoperative neurological injury

	OR	95% CI	*p* value
Female	0.90	0.67–1.22	0.491
Age (years)	0.97	0.91–1.04	0.397
ASA score (ref. ASA 1)			
2	0.99	0.72–1.34	0.985
3	1.37	0.90–2.08	0.142
4	6.43	2.24–18.45	< 0.001*
> 12 levels (ref. 7–12 levels)	1.02	0.78–1.35	0.865
Surgical year	0.98	0.92–1.04	0.475
Spinal navigation	0.51	0.28–0.94	0.030*

### Allogeneic transfusion, 30 day reoperation, and operative time

The rate of perioperative allogeneic transfusion for the entire study cohort was 11.9% (*n* = 2668). The rates of allogeneic transfusion for each surgical year from 2016 to 2022 are reported in Table 3. The rates ranged from 10.0 to 16.0%. The navigation cohort had a significantly lower rate of allogeneic transfusion (no navigation: 12.2% vs. navigation: 8.4%, *p* < 0.001) (Table 4). The NNT to prevent one allogeneic transfusion was 26. In the multivariable logistic regression analysis, the use of navigation was associated with reduced odds of allogeneic transfusion (OR 0.68 [95% CI 0.57–0.81], *p* < 0.001) (Table 7). Female sex (OR 1.64 [95% CI 1.47–1.84], *p* < 0.001), ASA score of 3 (OR 1.80 [95% CI 1.58–2.05, *p* < 0.001), ASA score of 4 (OR 7.63 [95% CI 4.50–12.95], *p* < 0.001), and > 12 surgical levels (OR 2.41 [95% CI 2.22–2.62], *p* < 0.001) were associated with higher odds of allogeneic transfusion. Increasing age in years (OR 0.95 [95% CI 0.93–0.97], *p* < 0.001) and subsequent surgical year (OR 0.94 [95% CI 0.92–0.96], *p* < 0.001) were associated with reduced odds of allogeneic transfusion.

**Table 7:** Multivariable regression analysis on spinal navigation and allogeneic transfusion

	OR	95% CI	*p* value
Female	1.64	1.47–1.84	< 0.001*
Age (years)	0.95	0.93–0.97	< 0.001*
ASA score (ref. ASA 1)			
2	0.95	0.85–1.05	0.287
3	1.80	1.58–2.05	< 0.001*
4	7.63	4.50–12.95	< 0.001*
> 12 levels (ref. 7–12 levels)	2.41	2.22–2.62	< 0.001*
Surgical year	0.94	0.92–0.96	< 0.001*
Spinal navigation	0.68	0.57–0.81	< 0.001*

The rate of 30 day reoperation for the entire study cohort was 1.4% (*n* = 312). The rates of 30 day reoperation for each surgical year from 2016 to 2022 are reported in Table 3. The rates ranged from 1.2 to 1.7%. There was no significant difference in 30 day reoperation between the two cohorts (no navigation: 1.4% vs. navigation: 1.5%, *p* = 0.564) (Table 4). In the multivariable logistic regression analysis, the use of navigation was not associated with odds of reoperation (*p* = 0.782) (Table 8). ASA score of 3 (OR 1.89 [95% CI 1.29–2.76, *p* = 0.001), ASA score of 4 (OR 3.73 [95% CI 1.13–12.38], *p* = 0.031), and > 12 surgical levels (OR 2.30 [95% CI 1.84–2.89], *p* < 0.001) were associated with higher odds of reoperation.

**Table 8:** Multivariable regression analysis on spinal navigation and 30 day reoperation

	OR	95% CI	*p* value
Female	0.86	0.66–1.12	0.260
Age (years)	1.00	0.95–1.07	0.899
ASA score (ref. ASA 1)			
2	1.33	0.98–1.81	0.067
3	1.89	1.29–2.76	0.001*
4	3.73	1.13–12.38	0.031*
> 12 levels (ref. 7–12 levels)	2.30	1.84–2.89	< 0.001*
Surgical year	1.01	0.95–1.07	0.764
Spinal navigation	1.06	0.72–1.56	0.782

The mean operative time for the entire cohort was 4.60 ± 1.52 h. The mean operative times for each surgical year from 2016 to 2022 are reported in Table 3. The mean operative time ranged from 4.47 h to 4.78 h. The spinal navigation cohort had a longer mean operative time (no navigation: 4.6 h vs. navigation: 5.0 h, *p* < 0.001) (Table 4).

## Discussion

This was an NSQIP Pediatric study including 22,384 patients who received posterior spinal fusion for idiopathic scoliosis. There were 1,879 patients in the navigation cohort. We found lower rates of postoperative neurological injury and perioperative allogeneic transfusion with use of navigation. We found no difference in the 30 day reoperation rate. Navigation was associated with increased operative time by a mean of 0.4 h. The use of navigation in pediatric idiopathic scoliosis increased from year 2016 to year 2022. To our knowledge, this is the largest study to date investigating the impact of navigation on perioperative outcomes in pediatric idiopathic scoliosis.

Pedicle screw deviation rates for freehand pedicle screw insertion ranges from 1.7 to 16% [7–11]. Previous studies have demonstrated reduced rates of pedicle screw breaches and improved accuracy with navigation [12–15]. Two meta-analyses reported an accuracy of 93–94% with navigation compared to 85% with freehand technique [16, 17]. Yu et al. reported 4.6% of screws breached the pedicles when placed with navigation compared to 16% with freehand technique [18]. In adolescent idiopathic scoliosis, Ughwanogho et al. reported the odds of a significant medial breach was 7.6 times higher without navigation and a screw was 8.3 times more likely to be removed intraoperatively in the non-navigated cohort [19]. Larson et al. reported a high accuracy rate of 99.3% with the use of navigation in congenital scoliosis [20]. A systematic review on pediatric spine surgery patients found screws placed using navigation were three times more likely to be deemed acceptable, twice as likely to be deemed “perfect”, and one-third as likely to be potentially unsafe compared to screws placed freehand [21]. Although navigation has been shown to improve accuracy and reduce screw deviation, it remains unclear whether it reduces neurological injury in idiopathic scoliosis surgery. A Scoliosis Research Society study on morbidity and mortality associated with spinal fusion for adolescent idiopathic scoliosis reported a 1.8% neurological complication rate from 2001 to 2003 [22]. Similarly, in our study we found a postoperative neurological injury rate of 1.2% in cases without navigation. We found that the use of navigation was associated with a significantly reduced rate of postoperative neurological injury, with a rate of 0.6%. The navigation cohort had a lower rate of postoperative spinal cord injury (no navigation: 0.30% vs. navigation: 0.05%) and a lower rate of nerve root/plexus/cauda equina injury (no navigation: 0.75% vs. navigation: 0.43%). Additionally, in our adjusted analysis, the use of navigation was significantly associated with reduced odds of neurological injury, likely related to improved pedicle screw placement, since its use is associated with both reduced spinal cord injury and nerve root injury. Neurological injuries associated with corrective forces during deformity correction are unlikely to be augmented by the use of navigation. Similar to our results, Shin et al., in a meta-analysis, reported no neurological injuries in 4814 screws inserted with navigation and three neurological injuries in 3725 freehand screws [17]. Conversely, a meta-analysis by Verma et al. reported no difference in neurological injury between navigated and non-navigated screws [16]. Kaur et al. reported a lower rate of any complication with navigation, but no significant difference in neurological complications [23]. The rate of postoperative neurological injury for pediatric idiopathic scoliosis surgery is low, but these events can be devastating when they occur. Our study found that 1 postoperative neurological injury was prevented for every 167 cases that used navigation (NNT = 167).

Surgery for idiopathic scoliosis can be associated with significant blood loss, requiring blood transfusion. The rate of transfusion for idiopathic scoliosis surgery ranges from 15 to 27%. [24–26] Allogeneic transfusion has been associated with negative outcomes including poor functional recovery, higher rates of postoperative infection, and higher rates of adverse events [27–29]. Therefore, it remains an important task to investigate strategies to minimize the use of allogeneic transfusion. Our study found a significantly lower rate of allogeneic transfusion in the navigation cohort (no navigation: 12.2% vs. navigation: 8.4%) and reduced odds of allogeneic transfusion with use of navigation. One allogeneic transfusion was prevented for every 26 cases that used navigation (NNT = 26). A study by Moore et al. investigated the use of navigation in posterior spinal fusion for adolescent idiopathic scoliosis using the NSQIP Pediatric database, years 2012–2018 [30]. Moore et al. reported a higher transfusion rate with navigation (no navigation: 65.0% vs. navigation: 75.6%) [30]. Interestingly, the transfusion rates reported by Moore et al. were much higher than the results from our study [30]. Similar to our results, previous studies have reported reduced rate of blood transfusion in adult lumbar spine surgery with use of navigation [31–33]. Hitti et al. reported reduced blood loss with the use of navigation for C1–C2 fusions [34]. A meta-analysis on the use of navigation for thoracic pedicle screw insertion reported lower intraoperative blood loss with navigation [35]. It is unclear how the use of navigation reduces the rate of allogeneic blood transfusion. One potential reason for reduced blood loss could be that the use of navigation requires less extensive soft tissue dissection for placement of pedicle screws, resulting in reduced blood loss [36]. In posterior spinal fusion surgery, the highest amount of blood loss occurs during the screw insertion stage [37]. While use of navigation has been associated with increased overall operative time, previous studies have reported reduced instrumentation time due to reduced intraoperative pedicle screw repositioning [35] and reduced time for insertion of each pedicle screw, which could contribute to reduced blood loss [38].

Previous single-center studies have reported reduced reoperation rates from misplaced pedicle screws with the use of navigation [39, 40]. A study by Schouten et al. investigating the role of navigation in acute spinal trauma found a lower rate of revision surgery for implant malposition with the use of navigation compared to freehand technique (0.0% vs. 1.2%) [39]. Watkins et al. reported that the rate of revision surgery for misplaced pedicle screws was reduced from 3 to 0% with the use of navigation [40]. However, in our study, we found no difference in the 30 day reoperation rate with the use of navigation (no navigation: 1.4% vs. navigation: 1.5%). Similarly, a large national database study by Kaur et al. found no difference in the 90 day reoperation rate with the use of navigation in adolescent idiopathic scoliosis surgery [23]. An NSQIP database study by Shuman et al. found no difference in 30 day reoperation with the use of navigation in adult spine surgery patients [31]. A possible explanation for the lack of difference in reoperation rates with the use of navigation is that instrumentation complications occurring at the time of surgery—such as cerebrospinal fluid leaks, neurological injury from malpositioned screws, unsafe pedicle screws, or large pedicle breaches—are likely addressed and corrected at the time of the initial procedure.

One common critique for the use of navigation is increased operative time due to the process of scanning and registration. Previous studies have reported longer operative time with the use of navigation, ranging from an average of 24–40 min of additional operative time [21, 30, 35]. In our study, we found longer operative time with the use of navigation, with a mean of 0.4 h. Interestingly, although the use of navigation is associated with increased total operative time, multiple studies have reported reduced time for screw insertion with navigation compared to the freehand technique [35, 38]. Aside from increased operative time, other concerns regarding the use of spinal navigation include cost and exposure to radiation. Navigation systems involve substantial initial investment and ongoing maintenance expenses. Additionally, most navigation systems utilize computed tomography images which results in increased radiation exposure for the patient. This is particularly a concern in pediatric patients, who are more sensitive to radiation than adult patients due to smaller body size, developing organs, and small amount of overlying tissue [41]. With the advent of magnetic resonance imaging-generated synthetic computed tomography, navigation without significant radiation exposure is a potential option in the future [42, 43]. However, further study on the use of magnetic resonance imaging-generated synthetic computed tomography for navigation is required.

The use of navigation in pediatric idiopathic scoliosis surgery remains understudied, despite the steady increase in use of navigation for pediatric spine surgery over the past decade. To our knowledge, this is the largest study investigating the impact of navigation on the rates of neurological injury, allogeneic transfusion, and reoperation in pediatric idiopathic scoliosis surgery. We found significantly reduced rates of neurological injury and allogeneic transfusion with the use of navigation. The results of our study demonstrate improved safety with pedicle screw placement and support the use of navigation in pediatric idiopathic scoliosis surgery. However, there are other factors to consider such as increased radiation exposure in pediatric patients, longer total operative time, and logistical challenges. There are several limitations to this study. This was a retrospective cohort study using administrative data. Therefore, there is selection bias that we are unable to control for. Additionally, there may be confounding variables that are relevant to our outcomes of interest that were not collected in the database, and, therefore, not included in our model. This includes important curve-related variables, such as curve magnitude, amount of rotation, stiffness of curve, and amount of curve correction. Additionally, the specific cause of postoperative neurological injury is not collected by the database. There could be postoperative neurological injury related to corrective forces during deformity correction. There are surgical factors such as use of tranexamic acid, ultrasonic bone scalpel, and use of hemostatic devices and agents that can impact transfusion rates, which we could not control for in our study. Although we attempted to control for confounders by isolating a more homogeneous patient population, there could be differences between the two study cohorts that are not adjusted for in our analysis. Furthermore, the NSQIP Pediatric database anonymizes the surgeon and institution. There are likely variations in surgeon practices and technique for navigation, as well as between-institution differences that we are unable to control for. Lastly, the use of navigation was determined using a CPT code and there are potentially cases in which navigation was used, but not coded for. Additionally, this code encompasses a variety of navigation techniques, including computed tomography navigation, fluoroscopy-based navigation, and robotic-assisted navigation, which may have inter-technique variability. Despite the limitations, results from our study add to the growing literature on use of navigation in pediatric spine surgery. The use of navigation in pediatric idiopathic scoliosis surgery significantly improves patient safety by reducing the risk of neurological injury and minimizing the need for allogeneic transfusion, underscoring its role in lowering overall morbidity and advancing the standard of care.

## Data Availability

The National Surgical Quality Improvement Program (NSQIP) Pediatric database is available for use by all participating NSQIP centers.
